# Exogenous Nkx2.5- or GATA-4-transfected rabbit bone marrow mesenchymal stem cells and myocardial cell co-culture on the treatment of myocardial infarction in rabbits

**DOI:** 10.3892/mmr.2015.3775

**Published:** 2015-05-12

**Authors:** PU LI, LEI ZHANG

**Affiliations:** 1 Department of Cardiac Surgery, The Third Hospital of Hebei Medical University, Hebei, Shijiazhuang 050017, P.R. China; 2 Department of Histology and Embryology, Hebei Medical University, Hebei, Shijiazhuang 050017, P.R. China

**Keywords:** bone marrow mesenchymal stem cells, cardiac differentiation, transfection, induction, Nkx2.5, GATA-4, myocardial infarction, cell transplantation

## Abstract

The present study aimed to investigate the effects of Nkx2.5 or GATA-4 transfection with myocardial extracellular environment co-culture on the transformation of bone marrow mesenchymal stem cells (BMSCs) into differentiated cardiomyocytes. Nkx2.5 or GATA-4 were transfected into myocardial extracellular environment co-cultured BMSCs, and then injected into the periphery of infarcted myocardium of a myocardial infarction rabbit model. The effects of these gene transfections and culture on the infarcted myocardium were observed and the results may provide an experimental basis for the efficient myocardial cell differentiation of BMSCs. The present study also suggested that these cells may provide a source and clinical basis for myocardial injury repair via stem cell transplantation. The present study examined whether Nkx2.5 or GATA-4 exogenous gene transfection with myocardial cell extracellular environment co-culture were able to induce the differentiation of BMSCs into cardiac cells. In addition, the effect of these transfected BMSCs on the repair of the myocardium following myocardial infarction was determined using New Zealand rabbit models. The results demonstrated that myocardial cell differentiation was significantly less effective following exogenous gene transfection of Nkx2.5 or GATA-4 alone compared with that of transfection in combination with extracellular environment co-culture. In addition, the results of the present study showed that exogenous gene transfection of Nkx2.5 or GATA-4 into myocardial cell extracellular environment co-cultured BMSCs was able to significantly enhance the ability to repair, mitigating the death of myocardial cells and activation of the myocardium in rabbits with myocardial infarction compared with those of the rabbits transplanted with untreated BMSCs. In conclusion, the exogenous Nkx2.5 and GATA-4 gene transfection into myocardial extracellular environment co-cultured BMSCs induced increased differentiation into myocardial cells compared with that of gene transfection alone. Furthermore, significantly enhanced reparative effects were observed in the myocardium of rabbits following treatment with Nkx2.5- or GATA-4-transfected myocardial cell extracellular environment co-cultured BMSCs compared with those treated with untreated BMSCs.

## Introduction

Heart disease is one of the most prevalent diseases affecting human health worldwide. Heart diseases, including acute myocardial infarction (AMI) result in a decreased number of surviving cardiac cells, scar tissue formation, ventricular remodeling, loss of myocardial contractility, decreased ventricular ejection fraction and eventually decreased heart function, which may induce life-threatening heart failure (1). Exogenous cell transplantation into the infarcted areas has been employed in order to induce these cells to perform the integrative biological functions, thereby potentially improving cardiac function (2). Over the past few decades, studies have investigated the use of cardiac stem cells in the repair of damaged heart muscle in order to restore heart function. Bone marrow mesenchymal stem cells (BMSCs) have been a predominant focus, which have numerous advantages compared with other stem cells, as they are a rich and extensive source of cells that are easily accessible. Furthermore, autologous transplantation may be performed without ethical issues. Culture and isolation methods for BMSCs are simple as they proliferate rapidly and have high amplification capability for culture *in vitro*, as well as gene stability following multiple passages. In addition, BMSCs have a high differentiation capacity and low immunogenicity. Therefore, BMSCs are ideal stem cells for transplantation in the treatment of cardiovascular disease.

There are three common methods of myocardial cell differentiation from BMSCs *in vitro*, which include drug induction, co-culture with myocardial cells and genetic modification (3). However, these methods are inefficient and therefore maximizing the efficiency of this process has been the focus of numerous studies.

Genetic modification of BMSCs may promote the transformation into cardiac cells at the molecular level. This novel method of differentiation is a result of development in molecular biology, which aims to activate cardiac differentiation genes and regulatory networks via the activation of one or more key genes in order to induce the transformation of BMSCs into cardiac cells (4). Early cardiac transcription factors are expressed in early heart development, the most important of which are Nkx2.5, GATA-4 and TBX5 (5–8). These transcription factors regulate the expression of numerous cardiac structural protein genes and promote BMSC differentiation into cardiomyocytes, which is a key step in normal heart development. Numerous studies have investigated the effectiveness of drug-induced differentiation of stem cells into cardiomyocytes; these drugs include 5-azacytidine (5-aza), dimethylsulfoxide (DMSO), insulin, dexamethasone and ascorbic acid (9–15). 5-Aza is the most commonly used drug to induce differentiation into cardiomyocytes; however, it has been reported to result in cell toxicity and therefore may not be appropriate for clinical treatment (16). At present, co-culture of BMSCs with cardiomyocytes is the most commonly used method of *in vitro* cardiomyocyte differentiation. It is also the simplest method, which is almost non-cytotoxic and therefore may be feasible for clinical use.

In the present study, early cardiac transcription factors Nkx2.5 or GATA-4 were transfected into BMSCs. In addition, myocardial cell co-culture methods *in vitro* were used in order to determine the effects of myocardial cell external culture on the differentiation capacity of BMSCs into cardiomyocytes. The present study therefore aimed to provide the experimental basis for the realization of efficient cardiac cell differentiation of BMSCs, as well as to provide an effective source and clinical basis for stem cell transplantation in myocardial cell injury repair.

## Materials and methods

### Isolation and culture of BMSCs in New Zealand white rabbits

One-week-old male and female New Zealand white rabbits (The Animal Experimental Center of Hebei Medical University, Shijiazhuang, China; weight, 200–250 g) were sacrificed via intraperitoneal injection of 10% chloral hydrate, and then placed into 75% ethanol for 5 min. Under strict sterile conditions, the humerus, femur and tibia were isolated and washed three times with 0.01 mol/l phosphate-buffered saline (PBS), containing 100 U/ml penicillin and 100 *µ*g/ml streptomycin (both North China Pharmaceutical Factory, Shijiazhuang, China). The epiphysis of the humerus, femur and tibia were then cut open and the bone marrow cavity was exposed. A 5-ml sterile syringe was used to wash the marrow cavity repeatedly with Dulbecco’s modified Eagle’s medium (DMEM)-F12 (Gibco-BRL, Grand Island, NY, USA). The cell suspension washed out was filtered using a 200-hole strainer and centrifuged at 174 × g for 5 min; the supernatant was then discarded. Cells were resuspended in complete cell culture medium and the single cell suspension was produced using 90 *µ*l cell suspension, which was added to 10 *µ*l trypan blue solution (0.4%; Beijing Cable Laibao Technology Co. Ltd., Beijing, China) for mixing and incubated for 5 min. An inverted microscope (AE2000; Motic Deutschland GmbH, Wetzlar, Germany) and a counting hemocytometer (Hebei Medical University, Shijiazhuang, China) were then used for counting. The study was approved by the ethics committee of Hebei Medical University, Shijiazhuang, China.

### Culture and subculture of BMSCs in New Zealand White rabbits

The isolated cells were seeded in 25-cm^2^ flasks (1×10^5^ cells/cm^2^) and then stored at 37°C and 5% CO_2_ in a humidified incubator thermostat (Shanghai Lishen Scientific Instrument Co., Ltd., Shanghai, China). The flasks were kept still and incubated for 48 h in order to promote cell adhesion. The media was then replaced every 2–3 days in order to remove non-adherent cells, until primary BMSCs formed. When the adherent cells were ~80% confluent, the culture medium was discarded and cells were washed 1–2 times with PBS, followed by the addition of 1 ml of 0.25% trypsin (Sigma-Aldrich, Shanghai, China) and 0.02% EDTA (Shanghaiziyi-Reagent, Shanghai, China) for digestion. The extent of digestion was observed under an inverted microscope. Total cell culture medium was added to terminate trypsin digestion when cytoplasmic retraction and increased cell gaps were observed; the digestion time was <2 min each time. Pipettes were used to remove the culture liquid from the culture flask in order to repeatedly and gently collect the cells at the bottom, air bubbles were avoided. Digested cells were made into a single cell suspension, and centrifuged at 174 x g for 5 min. The precipitated cells were then resuspended in total cell culture medium. Cells were passaged and inoculated into two flasks and incubated at 37°C and 5% CO_2_ using a humidified incubator thermostat for culture. The liquid was replaced every 2–3 days, when adherent cells reached ~80% confluence every 3–5 days and digestion was performed immediately. Passaging (1:2) was performed, passages were labeled as P1, P2 and P3. An inverted microscope was used to observe growth and the morphological characteristics of cells. P3 cells were selected for subsequent experiments.

### Co-cultured cardiomyocyte establishment in New Zealand white rabbits

Newborn New Zealand white rabbits (male and female; weight, 10–20 g), were sacrificed via intraperitoneal injection of 10% chloral hydrate. The hearts were isolated under strict aseptic conditions and washed three times with 0.01 mol/l PBS solution containing 100 U/ml penicillin and 100 *µ*g/ml streptomycin. The apex of the hearts were removed and sectioned into small pieces (1×1 mm), then placed into a sterile beaker containing 10 ml of 0.25% trypsin and 0.02% EDTA. Cells were then incubated in a water bath at 37°C with agitation for 5 min; a 5-ml sterile syringe was used to remove the storage solution in order to repeatedly wash the cells. The cell suspensions were then added to flasks containing DMEM-F12, which were stored on ice. This process was repeated until the apex became white floc; the DMEM-F12 liquid in flasks was filtered through a 200-hole strainer and the supernatant was discarded following centrifugation at 174 × g for 5 min. The total cell culture medium was used to resuspend precipitated cells and produce a cell suspension. The suspension was placed in sterile flasks and 10 *µ*l BRDU (Wuhan Boster Biotechnology, Ltd., Wuhan, China) was added. The flasks were incubated at 37°C and 5% CO_2_ in a humidified incubator thermostat. After 1 h, the culture liquid was placed into another culture flask for continued culture and the fluid was changed every 48 h.

### Identification of surface molecules on BMSCs from New Zealand white rabbits using flow cytometry

#### Cell obtainment

Well grown P3 cells were washed 1–2 times with PBS. Trypsin (0.125%) was then used to make the single cell suspension, which was centrifuged at 174 × g for 5 min. The cell pellets were collected for cell counting (1×10^6^ cells/tube), and washed twice with PBS, prior to centrifugation at 174 × g for 4 min. PBS (100 *µ*l) was then added to the cell precipitate. The cell suspension was transferred to a flow tube and divided at random into experimental group (n=3) and isotype control group (n=3).

#### Identification of surface molecules of BMSCs

Cells were divided into three experimental groups and three control groups. Each fluorescent antibody (5 *µ*l) was added to cells in each experimental group [PE-CD29 monoclonal antibody (BD Biosciences, Shanghai, China), PerCP/Cy5.5-CD90 monoclonal antibody (BioLegend, Shanghai, China), fluorescein isothiocyanate (FITC)-CD45 monoclonal antibody (BioLegend)] and each isotype control group [PE-immunoglobulin (Ig)M monoclonal antibody (BD Biosciences), PerCP/Cy5.5-IgG1 monoclonal antibody (BioLegend), FITC-IgG1 monoclonal antibody (BioLegend)]. Samples were then incubated at room temperature in the dark for 30 min. Flow cytometric analysis using a flow cytometer (Beckman Coulter, Inc., Brea, CA, USA) was then conducted in order to identify BMSC surface molecules.

### Exogenous gene transfection of Nkx2.5 or GATA-4, and co-culture with myocardial extracellular environment to induce the differentiation of BMSCs into myocardial cells

#### Experimental grouping

Cells in each group underwent transfection and co-culture with myocardial cells as follows:

A1 group, transfection of p-enhanced green fluorescent protein (EGFP)-N1-Nkx2.5
A2 group, transfection of pEGFP-N1-Nkx2.5 and myocardial cell co-culture
A3 group, blank culture group of BMSCs
B1 group, transfection of pVP22-GATA-4/myc-His
B2 group, transfection of pVP22-GATA-4/myc-His and myocardial cell co-culture
B3 group, blank culture group of BMSCs

#### Competent E. coli DH5α preparation

Competent *E. coli* were selected from single colonies of newly activated *E. coli* DH5α (Biovector, Co., Ltd., Beijing, China) and incubated in 3–5 ml LB liquid medium (Gibco-BRL) at 37°C and agitated overnight. When in logarithmic growth phase, the bacterial suspension was diluted 1:100 or 1:50 and inoculated and agitated in the 100 ml LB liquid medium at 37°C for amplification. When the suspension appeared cloudy, optical density 600 was measured every 20 or 30 min until it reached 0.3–0.5 and culturing was stopped.

The cells were then placed on ice for 20 min, and centrifuged at 4°C at 4,000 × g for 10 min. The supernatant was discarded and 10 ml CaCl_2_ (0.1mol/l) was added. The cells were then resuspended and placed on ice for 15–30 min. This process was repeated once.

For pcDNA3.1-TBX5 transformation, 200 *µ*l competent cells were placed on ice. pcDNA3.1-TBX5 was combined with 10 *µ*l deionized water and added to the cell suspension and placed on ice for 30 min. The cells were then heat shocked at 42°C in water for 90 sec and placed on ice immediately for 3–5 min. LB liquid medium (1 ml) was added and mixed gently and incubated at 37°C for 1 h.

The suspension was distributed on the LB plates (Amp~+), and cultured at 37°C. After 30–60 min, the culture dish was inverted and the cells were cultured at 37°C for 12–16 h. Single colonies from LB plates were selected and incubated in LB liquid medium containing Amp (3–5 ml) at 37°C, Glycerol (300 *µ*l) was added and cells were stored at −70°C. pcDNA3.1-TBX5 was then sequenced by Sangon Biotech (Shanghai) Co., Ltd (Shanghai, China). Plasmids were extracted using the PureLink^®^ HiPure Plasmid Midiprep kit (Thermo Fisher Scientific, Rockford, IL, USA).

pEGFP-N1 (negative control), pEGFP-N1-Nkx2.5, pcDNA3.1, pVP22-GATA-4/myc-His and pcDNA3.1-TBX5 (all Biovector Co., Ltd.) were added to LB plates and the plates were cultured upside down at 37°C for 12–16 h. Single colonies were selected and incubated in 25 ml LB liquid medium for 16–21 h. Plates containing pEGFP-N1 or pEGFP-N1-Nkx2.5 were incubated in the LB with Amp, and the plates containing pcDNA3.1, pVP22-GATA-4/myc-His and pcDNA3.1-TBX5 were incubated in the LB with Kana.

The bacteria were collected in 50-ml tubes and centrifuged at 10,000 × g for 10 min. The supernatant was discarded and 4 ml suspension buffer was added. Cell lysate (4 ml) was incubated at room temperature for 5 min. Buffer (4 ml) was added to stop cell lysis and the suspension was centrifuged at 12,000 × g for 10 min.

Samples were added to a purification column Wizard^®^ Plus Minipreps DNA Purification System, Promega (Beijing) Biotech Co., Ltd. (Beijing, China) after equilibrium. The solution was passed through the column and lysis solution was discarded. The column was washed twice with washing buffer, 10 ml each time. The column was washed with 5 ml elution buffer and the solution containing the plasmids was collected. The DNA was precipitated with 3.4 ml isopropanol and centrifuged at 12,000 × g at 4°C for 30 min. The DNA was washed with 5 ml of 70% ethanol, and centrifuged at 12,000 × g for 5 min, the supernatant was then discarded. The precipitation was then air dried for 10 min, and suspended in 120 *µ*l sterile water without nucleases. It was loaded into 1.5 ml EP tubes, the concentration was detected and the tubes were stored at −20°C.

DNA transfection. When P3 cells were grown to 80% confluency, they were selected and serum-free, antibiotic-free DMEM-F12 medium was used for passage. Cells (4×10^4^/cm^2^) were seeded into six-well plates and incubated for 24 h, until the cells reached 90–95% confluence; well-grown cells were then selected for subsequent transfection. Plasmid DNA (4 *µ*g) was diluted in 250 *µ*l serum-free, antibiotic-free DMEM-F12 medium. Lipofectamine™ 2000 reagent (Invitrogen Life Technologies, Carlsbad, CA, USA) was gently agitated prior to transfection and diluted in 250 *µ*l serum-free, antibiotic-free DMEM-F12 medium. Each solution was then incubated at room temperature for 5 min and combined (total volume, 510 *µ*l); following gentle agitation, they were incubated at room temperature for 20 min to form DNA-Lipofectamine 2000 complexes. This was repeated for cells in each group. Cells were centrifuged at 12,000 × g for 5 min and the supernatant was discarded. The transfected cells were then washed twice with serum-free antibiotic-free medium and then 500 *µ*l DNA-Lipofectamine 2000 complexes were added into each well. Following agitation, the cells were incubated at 37°C and 5% CO_2_ for 3–5 h, and then washed twice with PBS. Cells were then cultured in 10% fetal bovine serum (PAA Laboratories GmbH, Pashing, Austria) antibiotic-free DMEM-F12 medium for 48 h in order to allow cells to express the exogenous gene.

### Exogenous gene expression

#### Cell culture of exogenous gene-transfected BMSCs

All groups were incubated at 37°C in 5% CO_2_ in a humidified incubator for 4 weeks, during which time no passage occurred. An inverted contrast microscope (Olympus IX83, Olympus, Tokyo, Japan) was used to observe cell differentiation each day. Immunocytochemistry and western blot analysis were then used to detect specific cardiac troponin T (cTnT) and connexin 43 (Cx43) expression in myocardial cells in each group.

#### Immunocytochemistry

Cell specimens were cultured for 4 weeks in each group and the expression of cTnT and Cx43 was analyzed by the SP method (17). The specimens were incubated in 3% methanol-H_2_O_2_ at room temperature for 10 min. After washing three times for 5 min in 0.01 mol/l PBS, they were treated with 1% Triton X-100 and then incubated with 10% normal goat serum. Primary anti-cTnT (1:100; sc-20025; Santa Cruz Biotechnology Inc., Santa Cruz, CA, USA), primary anti-Cx43 (1:100; #3512; Cell Signaling Technology, Inc., Danvers, MA, USA) primary antibodies (dilution, 1:100) were added for incubation. In the negative control group the primary antibodies were replaced with PBS. Biotinylated goat anti-mouse IgG (1:100, BA1001, Boster Bio-engineering Co., Ltd, Wuhan, China) and goat anti-rabbit (1:100, BA1001, Boster Bio-Engineering Co., Ltd, Wuhan, China) secondary antibodies and horseradish peroxidase-labeled chain enzyme avidin fluid were then added and incubated. Then the samples were stained with 5% DAB (D8230–5; Beijing Solarbio Science and Technology Co., Ltd., Beijing, China) and hematoxylin, dehydrated in gradient alcohol, cleared with xylene and mounted with neutral gum. The image data were analyzed with Image-pro Plus 6 software (Media Cybernetics, Inc., Rockville, MD, USA). The integral absorbance (IA) value was measured (Beckman Coulter, Inc.) and averages were taken.

#### Western blot analysis

Cells were transfected for 48 h and washed twice in PBS that had been precooled at 4°C. Then, lysis buffer was added to each group and centrifuged to save the supernatant containing proteins. The proteins were separated by SDS-PAGE and transferred to polyvinylidene fluoride (PVDF) membranes (Hefei United-Bio Co., Ltd., Anhui, China) that had been pretreated with methanol. The primary antibodies, anti-Nkx2.5 (1:500; #8792; Cell Signaling Technology, Inc.), anti-Myc (1:500; #5605; Cell Signaling Technology, Inc.) and anti-TBX5 (1:500; sc-17866; Santa Cruz Biotechnology Inc.), were diluted and added to the groups A1, A2 and A3, and the anti-β-actin (1:1,000; #4967; Cell Signaling Technology, Inc.) antibody was added to groups B1, B2 and B3. The groups were then incubated at 4°C overnight. The membranes were washed three times for 5 min with TBST. The membranes were then incubated with secondary antibodies (horseradish peroxidase labeled goat anti-rabbit (1:1,000; #7074; Cell Signaling Technology, Inc.) and goat anti-mouse (1:1,000; #7076; Cell Signaling Technology, Inc.) at 37°C for 2 h. The PVDF membrane was placed into the X-ray film cassette and tablet (Thermo Fisher Scientific, Inc.) in the darkroom. The film was screened using a scanner (Epson (China) Co., Ltd. Beijing, China) and analyzed with BIO-1D software (GoldSim, Issaquah, WA, USA) and corrected to β-actin, which served as internal reference. The relative expression of protein was presented by the ratios of absorbance of target protein to the absorbance of β-actin.

#### Statistical analysis

SPSS 13.0 software (SPSS, Inc., Chicago, IL, USA) was used for statistical analysis and all values are expressed as the mean ± standard deviation. One way analysis of variance was used for comparison among groups followed by post hoc tests; when the homogeneity of variance was met, a least significant differences test was used and if it was not met, the non-parametric Tamhane’s T2 method was used. α=0.05 was taken as the standard, P<0.05 was considered to indicate a statistically significant difference between values.

### Effects of exogenous gene transfection of Nkx2.5 or GATA-4, and myocardial cells outer environment co-culture of New Zealand rabbit BMSCs on the repair of infarcted myocardium

#### Experimental grouping

Transfected cells co-cultured with myocardial cells and blank cells were injected into rabbits with induced myocardial infarction as follows:

A1 group, transfection of pEGFP-N1-Nkx2.5 and myocardial cell co-culture (n=10)
A2 group, blank group of BMSCs (n=10)
B1 group, transfection with pVP22-GATA-4/myc-His and myocardial cell co-culture (n=10)
B2 group, blank group of BMSCs (n=10)

#### Establishment of rabbit models with acute myocardial infarction

Left anterior descending coronary artery ligation was used to establish the acute myocardial infarction model, as previously described (18). Briefly, New Zealand white rabbits were anesthetized using an injection of 10% chloral hydrate (3.5 ml/kg) into the ear vein. Head and limbs were fixed onto a surgical board, and the neck and chest skin were disinfected using povidone-iodine. The trachea was then isolated in order to insert a tube for ventilation and the ventilator parameters were adjusted (expiration:inspiration, 1.5:1; respiratory rate, 30–35 beats/min); following connection between the trachea cannula and ventilator, positive pressure ventilation was performed. A sternal line vertical incision was used to cut open the skin of New Zealand white rabbits and a thoracotomy was performed in order to expose the heart. The pericardium was cut open and a sterile cotton swab was used to gently lift the heart, while the rabbits were rotated slightly to the right. Approximately 1–2 mm below the junction of the pulmonary cone and left atrial appendage, 4–0 non-damage ligation lines (Johnson (China) Investment Co., Ltd. Shanghai, China) were used to line the left anterior descending coronary artery and left ventricular anterior wall myocardial infarction was established. A pale left ventricular anterior was used as an indicator of successful myocardial infarction induction. The heart was then put back into the chest cavity and the heartbeat was restored for 1 min.

#### Mesenchymal stem cell transplantation of New Zealand white rabbits

Following establishment of the myocardial infarction model, rabbits were left until their breathing became regular and then a sterile cotton swab was used to gently lift the heart, while the rabbits were rotated slightly to the right. A 1-ml syringe was used to inject the prepared cell suspension into two points (50 *µ*l/point) of the left ventricular myocardium at the border zone of infarcted myocardium and normal myocardium, avoiding cell spillage. The heart was then put back, the muscle and skin were sutured, and the chest was closed immediately. In the event of cardiac arrest, the heart was massaged in order to restore heartbeat and non-assisted breathing. Following surgery, ventilator and oxygen support was provided; following the restoration of spontaneous breathing, the ventilator was removed and 30 min later, the oxygen was removed. An intraperitoneal injection of 25,000 U/kg penicillin was then administered in order to avoid infection.

### Tissue sampling, H&E staining and immunohistochemical staining

#### Perfusion sampling method

Chloral hydrate (10%) was used to anesthetize the New Zealand white rabbits through the auricular vein. The thoracic cavity was opened avoiding the transplantation site. Irrigation fluid was perfused into the left ventricle, a small incision was made in the right atrium and the blood and perfusate was allowed to flow out. Following perfusion of physiological saline, the blood was washed out and added to 200–400 ml of 4% paraformaldehyde. The hearts were removed immediately following perfusion and the scar area and slightly purple cell injection zone were identified; the tissues were fixed in 4% paraformaldehyde for 24 h. The tissues were then washed in 0.01 M PBS for 12 h, dehydrated with gradient ethanol (each step, 2 h incubation) and xylene, and embedded in paraffin. The paraffin block was sectioned using a Leica RM2125 paraffin machine (Leica Biosystems, Shanghai, China) for continuous paraffin sections with a thickness of 5 *µ*m.

#### H&E staining

The sections were dewaxed in xylene I, xylene II, 100% alcohol I and 100% alcohol II for 10 min, and then in 95% alcohol, 90% alcohol, 80% alcohol, 70%alcohol for 5 min, followed by a wash with distilled water. The sections were stained with hematoxylin for 1 min and washed with distilled water. Differentiation was conducted using 1% hydrochloric acid, followed by a wash in tap water. Slides were then counterstained with eosin for 2 min. Slides were then dehydrated in gradient alcohol, 70% alcohol, 80% alcohol, 90% alcohol, 95% alcohol for 2 min, follwed by 100% alcohol I and and 100% alcohol II for 10 min. They were then immersed in xylene for 1 min and covered with neutral gum.

#### Immunohistochemistry staining

Prior to dewaxing, the tissues were placed in 4% xylene and then in 20% sucrose solution at 4°C overnight. Tissues were then fixed in paraffin. The sections were incubated at 68°C for 20 min and dewaxed in xylene I, xylene II, and gradient alcohol. The sections were incubated in 3% H_2_0_2_ at 37°C for 10 min, then washed with PBS three times. The sections were boiled in citric acid buffer solution (0.01 M, pH 6.0) at 95°C for 15 to 20 min, cooled for 20 min, washed with cold water and then washed with PBS three times. Normal goat serum was added for 20 min. Anti-cTnT and anti-Cx43 primary antibodies were added and incubated at 4°C overnight, followed by three washes with PBS. Biotinylated goat anti-mouse IgG and goat anti-rabbit secondary antibodies were then added, followed by washing with PBS. Horseradish peroxidase-labeled avidin and fluid was added and incubated at 37°C for 30 min, followed by three washes with PBS. DAB was added for 5 to 10 min and slides were observed under an inverted microscope. Slides were then washed with distilled water three times and PBS was added to terminate the staining reaction. Slides were counterstained with hematoxylin for 2 min, differentiated with 0.1% hydrochloric acid and washed with water. They were then dehydrated in gradient alcohol, immersed in xylene for 1 min and covered with neutral gum.

#### Collagen staining

Tissues were fixed in 10% formalin solution for 1 h and dehydrated and embedded in paraffin. Dewaxed paraffin slices were hydrated and stained with Weigert iron hematoxylin for 5–10 min. Excess stain was removed by washing in water. Hydrochloric ethanol (1%) was added for differentiation, any excess was removed by washing in water. Samples were then stained with Van Gieson for 2 min. Samples were desiccated with 95% ethanol and put in a temperature box and dried. Samples were then dehydrated with ethanol, cleared in xylene and mounted with neutral gum. They were then analyzed with the AE2000 inverted microscope.

## Results

### Flow cytometric analysis of surface molecules detected in BMSCs

**Figure 1:**
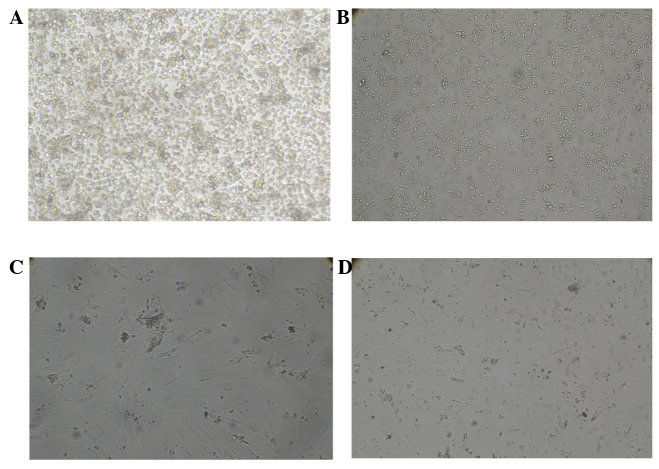
Primary New Zealand rabbit BMSCs were cultured with Dulbecco’s modified Eagles medium-F12 and images were captured at (A) 10 min and (B) 72 h following isolation (magnification, ×100). The third generation of rabbit BMSCs were cultured for 2 days and images were captured at magnification (C) ×100 and (D) ×400. BMSCs, bone marrow mesenchymal stem cells.

The successfully isolated cells were observed under an inverted microscope, they were found to be round or nearly round and were mixed with a large number of red blood cells and non-adherent mononuclear cells. Following 24 h, a small number of adherent cells were observed, which were round, polygonal or multilateral spindle shaped. Following 10 min, the non-adherent cells were removed and the number of adherent cells had markedly increased, the majority of which had a polygonal and spindle morphology, with a slow growth rate; the cells observed at this time point were the primary BMSCs (Fig. 1A). Following 2–3 days, adherent cells had rapidly proliferated and adjacent cells had gradually converged as agglomerates. In addition, some of the cell clusters were arranged radially (Fig. 1B). Following 6–7 days, the confluence of cells reached 80–90% and cells were arranged in a swirling or radial pattern. At this point, the primary passage was conducted. Following passage, BMSCs had more rapid adherent abilities than the primary cells. Within 24 h, all cells were adherent and demonstrated rapid, as well as evenly distributed, growth and proliferation. Following 3–5 days, 80–90% of cells were confluent adherent cells were and subsequent passage was then performed and cells were labeled as P1, P2 or P3. Over the three passages and medium replacements, non-adherent cells were gradually removed, round or oval cells decreased among adherent cells and adherent cell morphology gradually converged. When the passage reached the third generation, mesenchymal stem cells were predominantly in the same fusiform shape (Fig. 1C and D).

**Figure 2:**
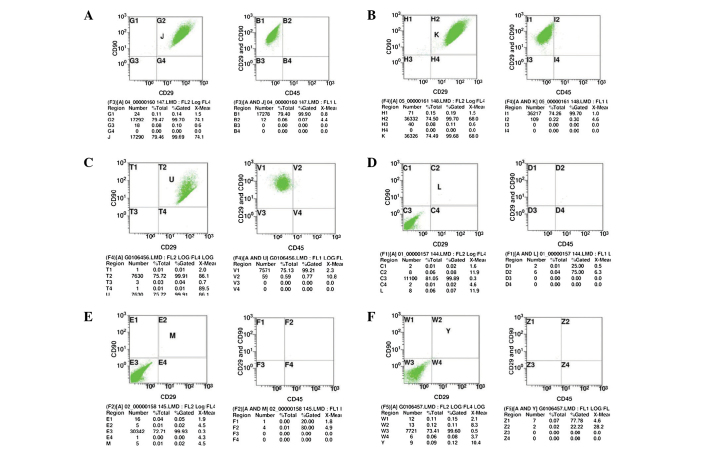
Flow cytometric analysis of New Zealand rabbit bone marrow mesenchymal stem cells labeled for cell-surface markers CD90^+^/CD29^+^/CD45^−^. The mesenchymal stem cells were grouped into (A-C) experimental group (n=3) and (D–F) control group (n=3). The experimental group was treated with 5 *µ*l fluorescent antibodies (PE-CD29, PerCP/Cy5.5-CD90, FITC-CD45) and the control group was treated with 5 *µ*l homotype fluorescent antibodies (PE-IgM, PerCP/Cy5.5-IgG1, FITC-IgG1), the cells were then incubated at room temperature in the dark for 30 min and flow cytometry was used to detect the surface molecules.

Flow cytometric analysis was used to detect the P3 cells with good growth. The results showed that the percentages of CD90^+^/CD29^+^ cells in the experimental groups (A1–3) were 99.70, 99.70 and 99.91%, respectively; and the percentages of CD45^−^ in CD90^+^/CD29^+^ cells were 99.90, 99.70 and 99.21%, respectively (Fig. 2A–C). The results suggested that in the experimental group (A1-3), the percentages of CD90^+^/CD29^+^/CD45^−^ cells were 99.61, 99.40 and 99.11%, respectively. The percentages of CD90^+^/CD29^+^ cells in the isotype control group (B1–3) were 0.08, 0.02 and 0.11%, respectively. Furthermore, the percentage of CD45^+^ in CD90^+^/CD29^+^ cells were 25.00, 20.00 and 77.78% (Fig. 2E–F). It suggested that in the experimental group, the percentages of CD90^+^/CD29^+^/CD45^+^ cells were 0.02, 0.00 and 0.11%. These results therefore indicated that the obtained mesenchymal stem cells were uniformly expressed and had high cell purities, which complied with the basic characteristics of BMSCs.

### Western blot analysis detection of exogenous gene expression

**Figure 3:**
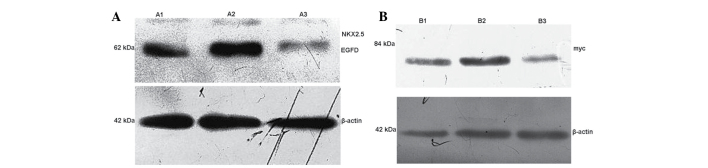
Western blot analysis of the expression of (A) Nkx2.5-EGFP in groups A1, A2 and A3 and (B) myc in groups B1, B2 and B3. EGFP, enhanced green fluorescent protein; A1, transfection of p-EGFP-N1-Nkx2.5; A2, transfection of pEGFP-N1-Nkx2.5 and myocardial cell co-culture; A3, blank culture of bone marrow mesenchymal stem cells; B1, transfection of pVP22-GATA-4/myc-His; B2, transfection of pVP22-GATA-4/myc-His and myocardial cell co-culture; B3, blank culture of bone marrow mesenchymal stem cells.

At 48 h post-transfection, the cellular total protein in A1, A2 and A3 groups were collected. Western blot analysis was used to detect the Nkx2.5-EGFP fusion protein expression in A1, A2 and A3 groups. The results showed that the exogenous expression of Nkx2.5 was observed following transfection in A1 and A2 groups, whereas NKx2.5 was not expressed in the control A3 group (Table I; Fig. 3A). The cell total protein was collected in groups B1, B2 and B3, and western blot analysis was used to detect Myc protein expression in each group. The results showed that exogenous GATA-4 expression was present following transfection in groups B1 and B2, whereas GATA-4 was not expressed in the control B3 group (Fig. 3B; Table II).

### Detection of cell differentiation of BMSCs following transgene induction

#### Cell morphology changes in New Zealand white rabbit BMSCs after transgene induction

**Figure 4:**
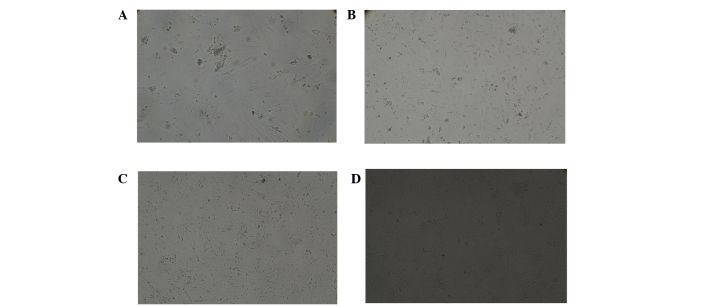
New Zealand rabbit BMSCs transfected with (A) Nkx2.5 (magnification, ×400) or (B) GATA-4 (magnification, ×200) and cultured for two weeks. New Zealand rabbit BMSCs transfected with (C) Nkx2.5 (magnification, ×200) or (D) GATA-4 (magnification, ×200) and co-cultured with heart cells for two. BMSCs, bone marrow mesenchymal stem cells. Images show the results of four samples taken from the same area.

Four weeks following transfection, inverted microscopy revealed that cells in the A1 and B1 groups were fusiform in shape; in addition, the growth of the cells showing overlay and high density features increased in a time-dependent manner (Fig. 4). When cell density reached a certain level, no further cell proliferation was observed and the morphological changes were similar in each group. The majority of aggregated myocardial cells in groups A2 and B2 were spindle shaped and certain cells with good growth showed a rhythmic beat. There was a high density of cells in groups A3 and B3 that were fusiform shaped, the growth of the local cells showed overlay and high density features.

#### Immunocytochemical analysis of BMSCs following transgene induction

**Figure 5:**
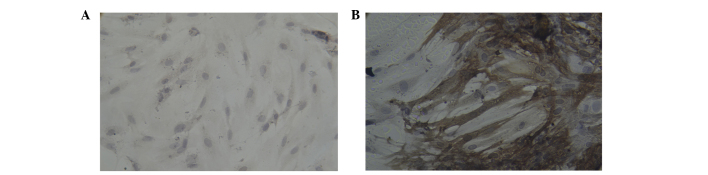
Immunocytochemical staining of the specific cardiac troponin T expression in groups (A) A1, BMSCs transfected with p-EGFP-N1-Nkx2.5 and (B) A2, BMSCs transfected with pEGFP-N1-Nkx2.5 and co-cultured with myocardial cells (magnification, ×200). BMSCs, bone marrow mesenchymal stem cells; EGFP, enhanced green fluorescent protein.

**Figure 6:**
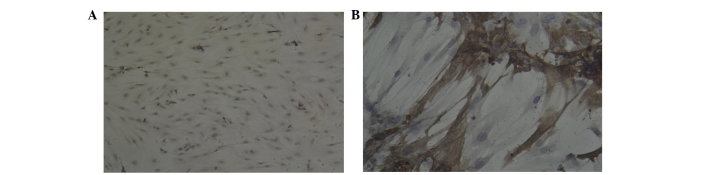
Immunocytochemical staining of the specific cardiac troponin T expression in groups (A) B1, BMSCs transfected with pVP22-GATA-4/myc-His (magnification, ×100) and (B) B2, BMSCs transfected with pVP22-GATA-4/myc-His and co-cultured with myocardial cells (magnification, ×200). BMSCs, bone marrow mesenchymal stem cells.

**Figure 7:**
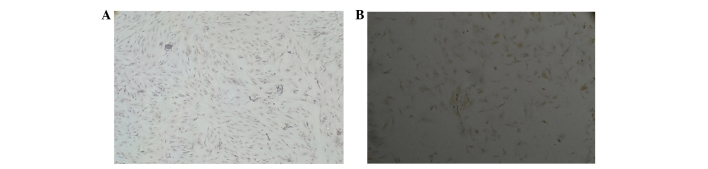
Immunocytochemical staining of the specific connexin 43 expression in groups (A) A1, BMSCs transfected with p-EGFP-N1-Nkx2.5 (magnification, ×100) and (B) A2, BMSCs transfected with pEGFP-N1-Nkx2.5 and co-cultured with myocardial cells (magnification, ×200). BMSCs, bone marrow mesenchymal stem cells; EGFP, enhanced green fluorescent protein.

**Figure 8:**
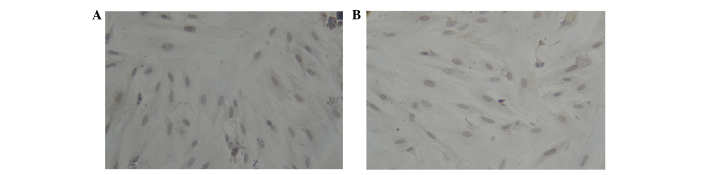
Immunocytochemical staining of the specific connexin 43 expression in groups (A) B1, BMSCs transfected with pVP22-GATA-4/myc-His and (B) B2, BMSCs transfected with pVP22-GATA-4/myc-His and co-cultured with myocardial cells (magnification, ×400). BMSCs, bone marrow mesenchymal stem cells.

**Figure 9:**
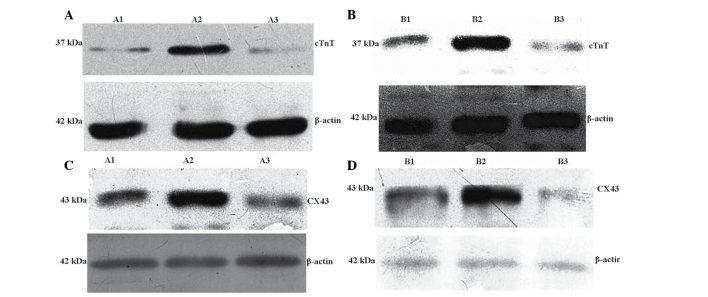
Western blot analysis of the expression of (A) cTnT in groups A1, A2 and A3, (B) cTnT in groups B1, B2 and B3, (C) Cx43 in groups A1, A2 and A3 and (D) Cx43 in groups B1, B2 and B3. cTnT, cardiac troponin T; Cx43, connexin 43; A1, BMSCs transfected with Nkx2.5; A2, BMSCs transfected with Nkx2.5 and myocardial cell co-culture; A3, blank culture of BMSCs; B1, BMSCs transfected with GATA-4; B2, BMSCs transfected with GATA-4 and myocardial cell co-culture; B3, blank culture of BMSCs; BMSCs, bone marrow mesenchymal stem cells.

Four weeks post-transfection and culture, immuno-cytochemical analysis revealed that certain cells were positive for cTnT in the A1 (Fig. 5A), A2 (Fig. 5B), B1 (Fig. 6A) and B2 (Fig. 6B) groups; brown mesh-like and particle-like structures were observed in the cytoplasm of cTnT positive cells. In addition, certain cells were positive for Cx43 in groups A1 (Fig. 7A), A2 (Fig. 7B), B1 (Fig. 8A) and B2 (Fig. 8B); brown particles were observed in the cytoplasm of Cx43-positive cells. However, few cells positively expressed cTnT and Cx43 in the A3 and B3 group. As shown in Table III, the integrating absor-bance value (IA) of each group demonstrated that in groups A1 and A2, cTnT and Cx43 expression levels were significantly increased following transfection of NKx2.5 compared with that of the blank transfected A3 group (P<0.05) ; with the highest expression levels observed in the A2 group (P<0.05) (Fig. 9A and C). In addition, cTnT and Cx43 expression levels were significantly increased in groups B1 and B2 following transfection of GATA-4, compared with that of the blank-transfected B3 group (P<0.05); furthermore, cTnT and Cx43 expression was significantly increased in the B2 group compared with that of the B1 group (P<0.05) (Table IV; Fig. 9B and D).

### Hematoxylin and eosin (H&E) and immunohistochemical staining of infarcted myocardium repair

#### Collagen fiber staining

**Figure 10:**
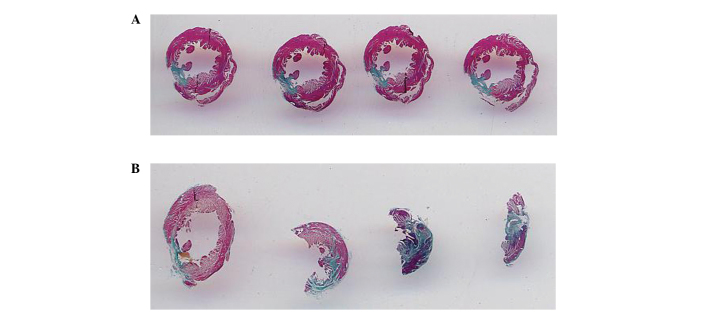
Staining for collagen. (A) Collagen fibers staining and (B) partial collage fiber staining in New Zealand myocardial infarction rabbit models. Pink staining, normal myocardium; blue-grey staining; infarct areas of the myocardium.

Following the establishment of a myocardial infarction rabbit model, collagen fiber staining was used in order to observe the effects of myocardial infarction model establishment on the heart. As shown in Fig. 10A and B, normal left ventricular myocardium was stained pink and myocardial infarct areas were stained gray-blue; these images demonstrated that the full left ventricular wall was stained gray-blue. Therefore it was concluded that the establishment of myocardial infarction models was successful and reached the requirement of experiments (Fig. 10A and B).

#### H&E staining

**Figure 11:**
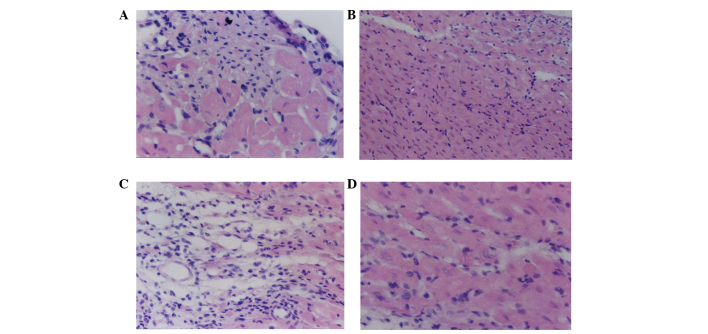
Hematoxylin and eosin staining of the myocardial infarction area of New Zealand rabbit myocardium *in vitro* following transplantation of BMSCs from groups (A) A1, transfected with Nkx2.5 and co-cultured with myocardial cells (magnification, ×200); (B) A2, blank BMSCs (magnification, ×100); (C) B1, transfected with GATA-4 and co-cultured with myocardial cells (magnification, ×200); and (D) B2, blanks BMSCs (magnification, ×400). BMSCs, bone marrow mesenchymal stem cells.

As shown in Fig. 11A and C, in the A1 and B1 groups, respectively, transplanted cells were observed to have survived and grown among the myocardial cells; in addition, these cells also showed radial and infiltrating growth into the surrounding area, where the transplanted cells at the junction between the transplanted area and the normal myocardial cell area gradually shifted to a spindle-shape. Fibroblasts and angiogenesis were observed and connections between cells were found at the junction between BMSCs and myocardial cells. In the A2 and B2 groups, transplanted cardiac cell differentiation and neovascularization were rarely observed (Fig. 11B and D); therefore indicating that the experimental effects of A1 and B1 treatments were markedly more beneficial compared with those of A2 and B2 (Fig. 11).

#### Immunohistochemical staining

**Figure 12:**
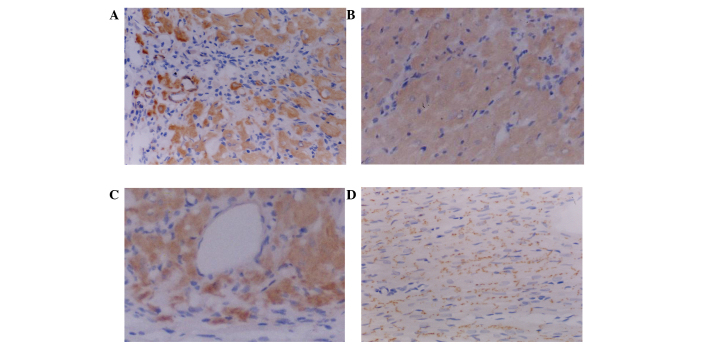
Immunohistochemical staining of connexin 43 expression in myocardial infarction area of New Zealand rabbit myocardium *in vitro* following transplantation of BMSCs from groups (A) A1, transfected with Nkx2.5 and co-cultured with myocardial cells (magnification, ×200); (B) A2, blank BMSCs (magnification, ×200); and (C) B1, transfected with GATA-4 and co-cultured with myocardial cells (magnification, ×400). (D) Immunohistochemical staining of connexin 43 in the B2 group, blank BMSCs (magnification, ×100). BMSCs, bone marrow mesenchymal stem cells.

Following transfected-BMSC transplantation in the A1 and B1 rabbit groups, immunohisto-chemical analysis revealed that the transplanted cells survived and grew among the myocardial cells (Fig. 12A and C). Brown stained substances were visible in the peripheral areas of the cells, which suggested that the transplanted cells successfully survive among myocardial cells or had been induced to differentiate into cardiomyocyte-like cells. Connections between myocardial cells were increased and the repair of damaged myocardial tissue was promoted. Certain brown-stained cells were able to self assemble in order to form vessel-like structures; in addition, to a certain extent, blood circulation within the damaged myocardium was increased and damaged heart muscle tissue repair was promoted. Transplanted cells showed overall growth of the transfected area as well as radial and infiltrating growth into the surrounding area. At the junction between transplanted cells and normal myocardium, the transplanted cells gradually shifted into spindle-shapes and the boundaries between the two areas became indistinguishable, where the brown staining was observed. This therefore indicated that the transplanted cells were able to partially or completely replace the function of the damaged portion of myocardial cells and novel cell connections were noticed between transplanted cells and myocardial cells. In A2 and B2 groups, the surviving transplanted cells stained brown and were distributed in the myocardium (Fig. 12B and D). These transplanted cells did not show signs of improving myocardial function; however, they showed improved cell connections between the myocardial cells. This therefore suggested that induced and myocardial cell co-cultured rabbit BMSCs had improved adaptive abilities in the myocardial microenvironment following transplantation compared with those of blank-transfected cells; therefore, indicating that these methods of BMSCs pre-treatment may induce the function of transplanted cells more rapidly and improve the survival of transplanted cells.

## Discussion

As a result of the rapid progress in the development of molecular biology techniques over recent years, gene transfection technology is currently used in various fields of biology. Gene transfection technology is used in order to transfer and deliver genes, which are then maintained and expressed in the cells. There are several types of transgenic plants and animals that are widely known to the public (18,20); however, gene trans-fection technology is also commonly used for gene therapy studies (21). In studies on the use of mesenchymal stem cell (MSC) therapy to repair damaged areas of the myocardium, MSCs transfected with particular genes were used to further improve the efficacy of bone marrow mesenchymal stem cell therapy. For example, certain studies have focused on improving the survival of BMSCs following transplantation, in which the heme oxygenase-1 (HO-1) was used to genetically modify BMSCs in order to enhance cell tolerance to hypoxia (22,23,24). In addition, angiogenin has been used to genetically modify BMSCs in order to protect against cardiomyocyte damage and promote the process of angiogen-esis. The overexpression of HO-1 or angiogenin in marrow MSCs was shown to increase the survival of transplanted cells and neovascularization, and improve cardiac function. Furthermore, insulin like growth factor, vascular endothelial growth factor, hepatocyte growth factor and Akt have been used to modify the gene expression of BMSCs (14–16,18,20, 25,26).

Gene transfection methods for eukaryotic cells are performed according to different mechanisms, which are divided into physical transfection methods, chemical transfection methods and viral transfection methods. Chemical transfection methods include the diethylaminoethyl cellulose (DEAE)-dextran method, the calcium phosphate method and the artificial liposome method (25–28); the most widely used method is the artificial liposome method (29). DEAE-dextran reagent was one of the earliest transfection reagents used in mammalian cells. It is a cationic polymer, which can be combined with negatively charged nucleic acids in order to be absorbed by the cell membrane (30). DEAE-dextran transfection is not reliable for stable transfection (30). The calcium phosphate co-precipitation transfection method is widely used in studies for transient and stable transfection as it is cheap and reagents are readily available. The method is as follows: DNA and calcium chloride are combined and then added to PBS in order to slowly form a DNA calcium phosphate precipite. The suspension containing the precipitate is then added to cultured cells and the DNA is uptaken by endocytosis of the cell membrane (18). In the artificial liposome method, the liposomes may also transport DNA and RNA *in vivo* in order to transfect into animals and humans for gene therapy; synthetic cationic liposomes bind with negatively charged nucleic acids to form a complex, which becomes surrounded by the cell membrane and is taken into the cytoplasm via endocytosis where the DNA complexes are released into the nucleus (31). Physical transfection methods include electroporation, microinjection and the gene gun method. The electroporation method involves high-voltage electroporation for interference of the cell membrane, so that pores are formed for easy entrance of nucleic acid into the cytoplasm. Using the electroporation method it is possible to inject millions of cells at a time, without using glass microin-jectors, technical training or expensive equipment. Compared with chemical transfection methods, electroporation has few biological or chemical side effects. As a physical method, elec-troporation is also less dependent on cell type, and is widely used and highly efficient (32). Microinjection methods are more expensive, but are effective for the induction of nucleic acid into human cells or nuclei. This method is used to produce transgenic animals; however, it is not suitable for studies that require a large quantity of transfected cells (33). The gene gun method uses high-speed particles to carry nucleic acids into cells; this method is applicable to cultured cells and cells in the body (34). The viral transfection method is an efficient gene delivery system; therefore, it is the most effective transfer method for the transfection of exogenous genes into a large number of cells in the human body. Viral infections have high efficiency, stable inheritance, may be applied to a variety of cells from different sources via simple methods, and can be conducted using retroviruses or adenoviruses. However, most viruses are potentially dangerous; therefore, operators are required to be experienced and have access high standard virus facilities (35). At present, the most widely used method of transfection is the artificial liposomes method as it has fewer issues compared with other methods. Lipofectamine 2000 is the third generation of multivalent cationic lipo-somes, which has significantly higher transfection efficiency, specificity and reproducibility compared with those of other liposomes. In addition, it is a non-immunogenic, non-infectious, non-carcinogenic and simple method for transfection. It has passed the authorization of NIH and recombinant DNA Advisory Committee (RAC) as a gene therapy vector for PhaseII clinical trials and has been used for the treatment of certain types of cancer (36). A previous study has shown that Lipofectamine 2000 successfully achieved the exogenous genes transfection of BMSCs (37). Therefore the present study used Lipofectamine 2000 for BMSC transfection. Western blot analysis revealed that liposomes successfully mediated plasmid transfection of pEGFP-N1-Nkx2.5 or pVP22-GATA-4/myc-His into BMSCs.

The specific mechanisms of cardiomyocyte differentiation of BMSCs *in vitro* remain to be elucidated. One of the primary aims of cardiomyocytes differentiation studies at present, is to identify the key genes and factors required to initiate the directed differentiation of BMSCs *in vitro* in order to cultivate a variety of cells and even organs. The present study investigated the transfection of Nkx2.5 and GATA-4, two closely associated transcription factors, which were reported to be involved in heart development (17,38). Nkx2.5 was found to participate in cardiac formation, including the right heart cyclization, differentiation of cardiac chambers, maturation of cardiac interval function, as well as the maintenance of the myocardium and the cardiac conduction system (39). GATA-4 has been shown to have an important role in cardiac proliferation, differentiation and survival as well as the cardiac hypertrophy process (40). The development process of the heart is not completed by a single or small number of genes, but a variety of genes which form regulatory networks, these genes include Nkx2.5 and GATA-4, which jointly regulate the normal development of the heart (41). At present, the gene regulatory mechanisms of heart development remained to be elucidated and require further investigation. The present study used the cationic liposome method to transfect Nkx2.5 or GATA-4 into BMSCs in order to determine whether the over-expression of transcription factors was able to induce BMSCs to differentiate into cardiomyocytes. Transfected BMSCs were co-cultured with cardiomyocytes in order to compare the effect of the two methods; previous studies on these methods are limited. The results of this experiment showed that the effect of co-culture in combination with transfection was more effective than transfection alone.

cTnT is a cardiac-specific troponin T, which is only expressed in the myocardium. In the present study, the results of the western blot and immunocytochemical analysis were consistent in the transfection only and transfection with co-culture groups, indicating that Nkx2.5 and GATA-4 upregulated the expression of cardiac-specific protein-cTnT following four weeks of culture.

Myocardial infarction animal models are commonly established using mechanical ventilation, chest coronary artery ligation or myocardial freezing injury (42–44). The myocardial freezing injury model forms identical infarct sizes as those of transmural myocardial infarction and may therefore be easily compared and observed; however, controlling the freezing time in the method is difficult. Freezing for too long may result in destruction of the cardiac tissue, whereas freezing for not long enough prevents effective transmural myocardial infarction; control of freezing time is particularly difficult in small animals, including mice and rabbits, due to the small volume of the heart. Therefore, this method is not commonly used (45–49). In the present study, coronary artery ligation was used to establish a rabbit myocardial infarction model as the cardiac structure of rabbits and humans are similar. The clinical and pathological mechanisms underlying coronary artery ligation in order to establish myocardial infarction in rabbits were similar to that of the mechanisms of myocardial infarction. This method is cheap and the surgical procedure is simple, with high success rates when performed by skilled surgeons; however, the disadvantage of this method is different infarct sizes due to the size of different animals. Human intervention cannot control the size of the infarct area following myocardial infarction; therefore, the efficacy has not been well observed. In the present study, the transplanted New Zealand rabbit BMSCs grew and survived among the myocardial cells. Cells were found to accumulate in the central transplantation area as a mass; however, they also demonstrated radial infiltrative growth in the peripheral transplant area. The cells at the junction between the transplanted area and normal myocardium gradually changed to a spindle shape. In addition, novel cell connections were formed between the transplanted cells and the rabbit myocardial cells, indicating that this connection may cause the reactivation of myocardial cells within the infarction area, which may restore the basic function of myocardial cells in the infarct area. In addition, in the infarct area, an increase in the number of fibroblasts and in angiogenesis was observed, which may have limited the continuous expansion of the infarct area and the impact of myocardial infarction on heart function. Novel blood vessel formation may have also improved the blood microcirculation and cellular microenvironment within the infarcted myocar-dium, which may increase the survival of myocardial cells. In addition, angiogenesis may increase the nutritional supply to transplanted exogenous cells and re-activate cardiac cells, therefore increasing their effectiveness. The differentiation and angiogenesis in the transfected BMSCs and co-cultured myocardial cell group was markedly increased compared with that in the control group. A degree of cardiomyocyte differentiation and angiogenesis were observed in the control group; however, these effects were improved in rabbits treated with BMSCs and co-cultured myocardial cells following transfection.

The results of the present study showed that following transplantation into areas with myocardial cells, the growth and survival of the transplanted cells was observed; immu-nohistochemical staining revealed brown-stained substances at the periphery of certain cells, suggesting that transplanted cells successfully survived among the myocardial cells or certain transplanted cells had been induced to differentiate into cardiomyocyte-like cells. Connections were observed between the myocardial cells, and certain cells with brown-staining were able to self-assemble to form vessel-like structures, which resulted in increased blood circulation within the damaged myocardium. In addition, the repair of damaged myocardial tissues was promoted in transplanted cell region and transplanted cells grew as a mass, as well as radial infiltrating growth into the surrounding area. The transplanted cells at the junction between the transplanted area and the normal myocardium area gradually shifted towards a spindle-shaped morphology and the boundaries between the two regions became indistinguishable. In addition, immunohistochemical staining indicated that the transplanted cells were able to partially or completely restore the function of the damaged portion of the myocardium. Novel cell connections were observed between the transplanted cells and myocardial cells, which suggested that transfection and co-culture of BMSCs prior to transplantation increased the adaptive ability of cells to the myocardial microenvironment as well as improved cell survival, compared with that of the control BMSCs. The results of the present study were only observed by microscopy. Further studies are required in order to determine the actual experimental results and survival rates of transplanted cells, as well as their role in the cardiac microenvironment.

In conclusion, the exogenous gene transfection of Nkx2.5 or GATA-4 in combination with myocardial extracellular environment co-culture of BMSCs, resulted in improved myocardial cell differentiation compared with that of gene transfection only. In addition, exogenous gene transfection of Nkx2.5 or GATA-4 into myocardial cell extracellular environment co-cultured BMSCs was able to enhance the ability to repair, mitigate the death of the myocardial cells and activate myocardium in myocardial infarction-induced rabbits.

## Figures and Tables

**Table I:** Western blot analysis was used to detect the expression of Nkx2.5-EGFP in cells of groups A1, A2 and A3 group.

Relative protein expression	A1 group	A2 group	A3 group
Nkx2.5-EGFP/β-actin	0.180±0.029^b^	0.184±0.031^a^	0.001±0.000^a^

Values are presented as the mean ± standard deviation.

a P<0.05 vs. the A1 group and

b P>0.05 vs. the A2 group. EGFP, enhanced green fluorescent protein; A1, transfection of p-EGFP-N1-Nkx2.5; A2, transfection of pEGFP-N1-Nkx2.5 and myocardial cell co-culture; A3, blank culture of bone marrow mesenchymal stem cells.

**Table II:** Western blot analysis was used to detect the expression of myc in cells of groups B1, B2 and B3 group.

Relative protein expression	B1 group	B2 group	B3 group
Myc/β-actin	0.036±0.040^b^	0.038±0.039^a^	0.000±0.000^a^

Values are presented as the mean ± standard deviation.

a P<0.05 vs. the A1 group;

b P>0.05 vs. the A2 group. B1, transfection of pVP22-GATA-4/myc-His; B2, transfection of pVP22-GATA-4/myc-His and myocardial cell co-culture; B3, blank culture of bone marrow mesenchymal stem cells.

**Table III:** Immunocytochemical staining and western blot analysis of cTnT and Cx43 expression in groups A1, A2 and A3.

Antibody	IA		
	A1 group	A2 group	A3 group
cTnT	0.226±0.051^b^	0.231±0.050^a^	0.155±0.006^a^
Cx43	0.271±0.030^b^	0.280±0.031^a^	0.176±0.024^a^
cTnT/β-actin	0.490±0.111^b^	0.498±0.098^a^	0.009±0.001^a^

Values are presented as the mean ± standard deviation.

a P<0.05 vs A1 group;

b P>0.05 vs A2 group. cTnT, specific cardiac troponin T; Cx43, connexin 43; A1, BMSCs transfected with Nkx2.5; A2, BMSCs transfected with Nkx2.5 and myocardial cell co-culture; A3, blank culture of BMSCs; BMSCs, bone marrow mesenchymal stem cells. IA, integral absorbance.

**Table IV:** Immunocytochemical staining and western blot analysis of cTnT and Cx43 expression in groups B1, B2 and B3.

Antibody	IA		
	B1 group	B2 group	B3 group
cTnT	0.222±0.041^b^	0.235±0.046^a^	0.155±0.006^a^
Cx43	0.262±0.022^b^	0.275±0.029^a^	0.176±0.024^a^
cTnT/β-actin	0.410±0.124^b^	0.437±0.101^a^	0.039±0.010^a^

Values are presented as the mean ± standard deviation.

a P<0.05 vs B1 group;

b P>0.05 vs B2 group. cTnT, specific cardiac troponin T; Cx43, connexin 43; B1, BMSCs transfected with GATA-4; B2, BMSCs transfected with GATA-4 and myocardial cell co-culture; B3, blank culture of BMSCs; BMSCs, bone marrow mesenchymal stem cells. IA, integral absorbance.
